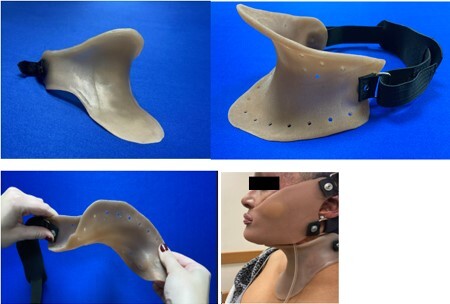# 775 A Unique Semirigid Silicone Neck Collar for Management of Hypertrophic Scars

**DOI:** 10.1093/jbcr/irae036.316

**Published:** 2024-04-17

**Authors:** Michelle N Dwertman, Catherine Freeman

**Affiliations:** University of Cincinnati Burn Unit, Cincinnati, OH; University of Cincinnati Burn Unit, Cincinnati, OH

## Abstract

**Introduction:**

In the realm of burn injuries, the use of rigid hard neck collars, or soft collars, is indispensable for optimizing neck positioning and scar management. However, when injuries encompass the head and neck, a critical challenge arises only one rigid orthosis can typically be worn one at a time. We are introducing a novel solution: a semirigid neck collar crafted from flexible silicone. This collar combines the benefits of semirigid support with the freedom of natural neck and allows jaw movement and may be worn with a rigid facemask. Furthermore, it can be customized to effectively manage hypertrophic scarring, making it a promising advancement in burn injury care.

**Methods:**

Our semirigid neck collar is crafted with High Consistency Rubber (HCR) silicone. The HCR can be fabricated as a solid sheet or perforated. This HCR applies a gentle, consistent minimum pressure of 20mmHg as displayed by the Kikuhime pressure monitoring device while remaining pliable enough to accommodate natural neck movements and TMJ mobility. The material is mixed, pigment tinted, and rolled to achieve the desired thickness. Following this, the HCR silicone is heated and molded based on a 3D scan of the patient's neck and fit to the patient.

A successful trial was conducted in a patient with severe facial and neck burns, resulting in improved neck ROM and hypertrophic scar progression. The resulting scar displayed improvement on the Vancouver Scar Scale yielded as reported on the Likert scale and the patient reporting increased compliance of the semirigid collar.

**Results:**

In the trial involving a patient with severe neck and facial burns:

1. Improved Neck ROM, Eating, and Mandibular Movement: While using the semirigid silicone neck collar, the patient experienced improvements in neck ROM, eating comfort, and mandibular movement. The average Likert scale rating for these improvements was five out of five.

2. Decreased Scarring upon Vancouver Scar Scale displaying the difference between the initial and final VSS scores, indicating how much the scar improved.

3. Patient Preference: A preference for the semirigid silicone neck collar, upon rating it a perfect five on the Likert scale. The patient was able to wear it in combination with a hard TFO facial orthotic.

**Conclusions:**

The semirigid silicone neck collar represents a move forward in the field of neck orthosis design, particularly concerning scar management. Its innovative blend of design elements, including flexibility, adaptability, and customizable features, holds great promise for individuals dealing with burn-related neck scarring.

**Applicability of Research to Practice:**

This semirigid appliance offers increased comfort compared to traditional rigid collars and an option in the management of neck positioning and scar hypertrophy. Its flexible nature, including color-tinting, and cost-effective modification options make it a valuable tool for improving scar management outcomes neck for patients with neck and combined facial/neck scarring.